# Railroad Surgeon at Cut Rates for Service

**Published:** 1892-04

**Authors:** 


### RAILROAD SURGEONS AT CUT RATES FOR SERVICES.

The medical profession, in this age of progress, is not only wide awake to advancement in knowledge, but is actuated by an *esprit du corps* which frowns upon anything which detracts from the high standard of character which has hitherto marked its attitude before the public. All competition not based upon merit should be discarded from the plans adopted in the race for preferment, and no respectable practitioner should entertain a proposition to do service at less than the recognized rates of the locality in which his lot is cast. By so doing, he not only lowers himself in the estimation of all honorable members of the profession, but does great injustice to others who are competitors for the patronage of the people.

It may be that one occupying a position as Railroad Surgeon cares nothing for these things so long as the money comes into his pocket. In this case an appeal to his pecuniary interest is the best mode of approaching him. It is evident that if all who desire employment in this line shall be firmly united in refusing positions except upon a regular basis of compensation, the authorities of the railroad companies must come up to the requirements. Will there be found men amongst us with such a craven spirit as to undertake the duties of a railroad surgeon at any price which may be fixed by the officials? If so, it may soon reach a point that work of all kinds, at all times and under all circumstances, will be imposed upon them for a mere pittance and the privilege of free passes over the roads. At present the schedule of rates enforced by some railroad companies is barely sufficient to meet the expenses incurred by the professional employe, and doubtless there are instances in which those who are called away from their private patients incur absolute loss in the discharge of their obligations to the company employing them. We hear some one say that I will take care of my own interests in this matter, and don't need any suggestions from others who cannot secure such a position. It is, doubtless, in the estimation of such an one, another case of sour grapes which actuates the criticism upon his course, and the complaint is thought to arise from inability to secure employment in this line of service. If such a feeling prompted this protest against the degradation of the profession, the writer would be amenable to the charge of envy or jealousy towards his colleagues. But the fact of his occupying an independent attitude enables him to speak out without prejudice in regard to an evil which is rapidly sapping the self-respect of every professional employee of railroad companies. Not only are poorly compensated services to the injured exacted, but the medical attendant is expected to favor the road in his testimony in an action for damages, and upon failure to do so, is liable to be dropped. An indignant representative of the interests of a railroad company retorts that no fee could induce him to give false testimony in a court of justice. But we must remember that facts have many shades of coloring for a medical witness, and the presumption is always greatly in favor of leaning to the side of the employer, and especially when the further connection of the witness with a railroad depends upon his turning a blind eye to whatever militates against its interests. All who have heard the testimony of railroad surgeons in damage suits will appreciate the awkward attitude in which he is placed under such circumstances.

An independent attitude, with regular rates of compensation for services rendered to railroad companies by the members of the medical profession, will do much towards relieving the position of its embarrassment.

There are grave questions of ethics involved in the existing relations of railroad surgeons, which do not come within the scope of this remonstrance against accepting positions without proper compensation.

It is intended to treat this purely as a business matter, and to urge upon all concerned strenuous resistance to the encroachments upon the rights and standing of otherwise worthy members of the profession.

The railroad companies cannot afford to employ an inferior man on account of his low price for services, and they should be made to understand that a good man cannot be had at a discount from regular rates.

